# Clinical analysis of the development of Guillain-Barré syndrome during bortezomib treatment for multiple myeloma

**DOI:** 10.3389/fneur.2025.1641892

**Published:** 2025-12-03

**Authors:** Jing-Gang Li, Chuan-Lei Chen, Da-Ji Liu, Zhi-Hong Zheng, Shu-Peng Liu, Shao-Yuan Wang, Xiang-Lei Chen

**Affiliations:** 1 Fujian Institute of Hematology, Fujian Provincial Key Laboratory on Hematology, Fujian Medical University Union Hospital, Fuzhou, China; 2 Department of Neurology, Yidu Central Hospital of Weifang, Qingzhou, China; 3 Department of Intensive Care Medicine, Yidu Central Hospital of Weifang, Qingzhou, China; 4 Electromyography Laboratory, Department of Neurology, Yidu Central Hospital of Weifang, Qingzhou, China; 5 Department of Hematology, Yidu Central Hospital of Weifang, Qingzhou, China

**Keywords:** bortezomib, GBS, multiple myeloma, immune treatment, prognosis

## Abstract

**Background:**

The development of Guillain-Barré syndrome (GBS) during bortezomib treatment for multiple myeloma (MM) is rare. Clinical vigilance regarding this serious adverse event is imperative for timely diagnosis and management.

**Methods:**

We conducted a retrospective review of the hospital’s health information system (HIS) and report three cases of GBS that occurred during bortezomib-based treatment in patients with MM. A retrospective analysis of the patients’ clinical presentations, diagnostic processes, treatments, and prognoses was conducted. Additionally, a literature search was performed by using the keywords “multiple myeloma,” “bortezomib,” “GBS,” and “polyneuropathy” in the PubMed and China National Knowledge Infrastructure (CNKI) databases, which yielded 30 relevant published cases for review.

**Results:**

A total of 33 cases were included in the analysis. The VRD regimen (bortezomib, lenalidomide, and dexamethasone) appeared to be associated with the development of GBS in patients with IgA-type MM, whereas the VTD regimen (bortezomib, thalidomide, and dexamethasone) was more commonly associated with IgG-type MM. Intravenous immunoglobulin (IVIG) and plasma exchange represented the main first-line treatments, and most of the patients achieved varying degrees of neurological recovery within a median of 4.5 months (range: 3 weeks to 21 months).

**Conclusion:**

Neurological examination, cerebrospinal fluid analysis revealing albuminocytologic dissociation, and nerve conduction studies were helpful for diagnosing GBS. Prompt treatment with IVIG and/or plasma exchange can significantly improve patient outcomes.

## Introduction

Multiple myeloma (MM) is a malignant clonal plasma cell disorder characterized by the proliferation and infiltration of clonal plasma cells in the bone marrow, elevated levels of monoclonal immunoglobulins in the peripheral blood, anemia, bone pain, and vertebral compression fractures. The widespread use of novel therapeutic agents (particularly proteasome inhibitors such as bortezomib) has significantly improved patient outcomes (1). However, the occurrence of adverse effects such as peripheral neurotoxicity often necessitates dose reduction or even discontinuation of bortezomib therapy.

Guillain-Barré syndrome (GBS) is an immune-mediated acute inflammatory peripheral neuropathy demonstrating heterogeneous clinical manifestations. GBS typically presents as flaccid, ascending paralysis and may be accompanied by autonomic dysfunctions such as urinary retention (2). GBS usually follows a monophasic course, peaking within 2–4 weeks. Plasma exchange and intravenous immunoglobulin (IVIG) therapy are the mainstays of treatment, and varying degrees of neurological recovery can be expected within weeks to months after treatment initiation.

Bortezomib-induced peripheral neuropathy (BIPN) predominantly affects sensory nerves, although case reports have described motor nerve involvement and GBS-like presentations (3). In this study, we retrospectively analyzed three cases of newly diagnosed MM patients who developed GBS during bortezomib-based treatment, and we reviewed the relevant literature to explore the potential mechanisms underlying GBS in the context of bortezomib therapy. Informed consent was obtained from all of the patients.

## Methods

### Case identification by hospital information systems and diagnostic criteria for GBS

A search was conducted using the Hospital Information System (HIS), with a focus on diagnostic codes obtained from the International Classification of Diseases, 10th Revision (ICD-10), specifically within the range of G60–G64, which encompasses peripheral neuropathies and related disorders. Between January 2003 and February 2025, 1 patient was identified in the Department of Hematology at Fujian Medical University Union Hospital, and 2 patients were identified in the Department of Hematology at Yidu Central Hospital of Weifang. All 3 patients were diagnosed with MM and developed GBS during bortezomib treatment. The diagnostic criteria (2) for GBS in 3 patients were determined according to the guidelines of the European Academy of Neurology (EAN) and the Peripheral Nerve Society (PNS), in conjunction with the Brighton criteria (4).

### Systematic literature review

**Figure 1:**
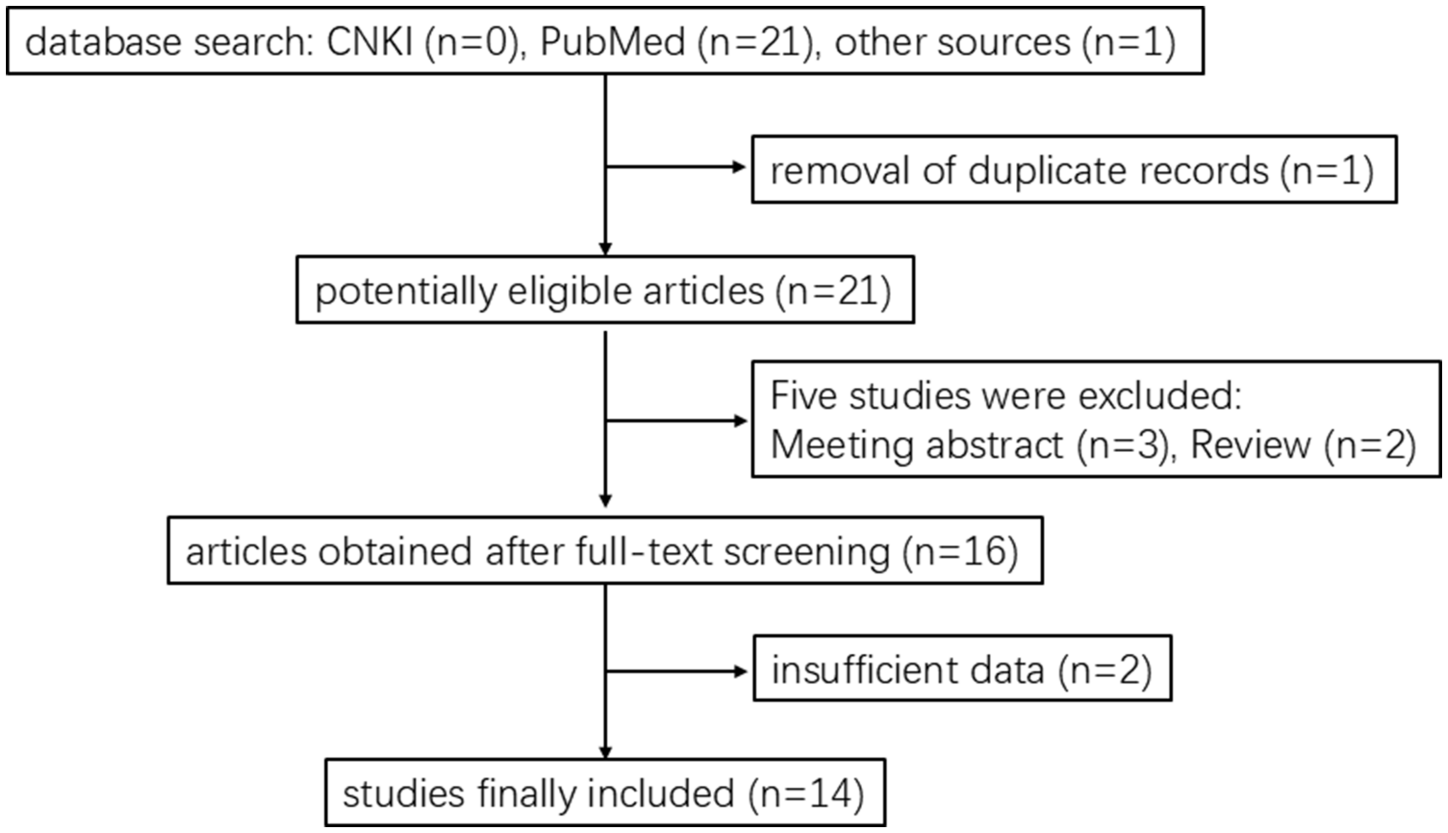
Flowchart of study selection process. Initially, 22 records were identified: 0 from CNKI, 21 from PubMed, and 1 from other sources. After removing 1 duplicate, 21 articles were potentially eligible. Five studies were excluded: 3 meeting abstracts and 2 reviews, leaving 16 articles after full-text screening. Two were excluded for insufficient data, resulting in 14 studies included.

A comprehensive literature search was performed in databases such as the PubMed and China National Knowledge Infrastructure (CNKI) databases using the keywords “multiple myeloma,” “myeloma,” “bortezomib,” “Guillain-Barré syndrome,” “polyneuropathies” and “polyneuropathy.” The search period was established from January 2003 to February 2025. The inclusion criteria were as follows: (1) studies including patients diagnosed with MM who developed GBS during bortezomib treatment; and (2) article types including original research, case reports, or case series. The following exclusion criteria were utilized: (1) studies including patients in whom GBS developed prior to the initiation of bortezomib therapy; and (2) conference abstracts for which the full text was not available. The literature screening process is summarized in Figure 1.

## Results

### Case presentation

#### Case 1

A 66-year-old male presented to Fujian Medical University Union Hospital in December 2022 with a complaint of bone pain. He was diagnosed with MM (IgG-kappa subtype, ISS stage III, R-ISS stage III). The patient received one cycle of the VRD regimen (bortezomib, lenalidomide, and dexamethasone), after which his bone pain improved. In February 2023, he developed watery diarrhea after receiving the first dose of the second cycle of bortezomib in the outpatient setting. The diarrhea resolved following symptomatic treatment. However, he subsequently experienced numbness and pain in the extremities, muscle weakness, abdominal distension, and difficulty urinating. These symptoms were accompanied by dizziness and hyperhidrosis. Supportive treatment with neurotrophic agents and pregabalin was administered; however, the patient’s symptoms worsened. His muscle strength decreased to grades 1–2 in both proximal and distal muscles of all four limbs, with normal muscle tone being noted. Sensory examination revealed reduced pain and tactile sensation below the umbilicus. Proprioception was intact, with joint position, movement and vibration sense preserved in both upper and lower limbs; assessment of balance and Romberg test could not be performed due to severe muscle weakness. Deep tendon reflexes, including biceps, triceps, patellar, and achilles tendon reflexes, were absent. On neurological examination, there was no evidence of muscle atrophy or fasciculations. Babinski sign was negative bilaterally, indicating intact upper motor neuron function. Spinal MRI revealed the following changes: (1) compressive changes in the T5, T7, and L1 vertebrae; (2) heterogeneous signals in the cervicothoracic spine; and (3) nodular lesions in the T10 and T12 vertebrae. On the seventh day after symptom onset, nerve conduction studies (NCSs) demonstrated the symmetric involvement of both motor and sensory fibers, along with mild motor fiber impairment, prominent sensory fiber involvement, and predominant axonal damage combined with demyelinating features. The detailed results of the nerve conduction studies (NCS) are provided in Supplementary Table 1. Needle electromyography (EMG) examination (Supplementary Table 2) showed reduced voluntary recruitment, consistent with impaired neural activation rather than primary myogenic damage. Cerebrospinal fluid (CSF) analysis revealed albuminocytologic dissociation, with an elevated protein concentration of 1,202 mg/L (150–450 mg/L) and a normal cell count of 4 cells/μL (0–5 μL). The serum antibody test result was positive for anti-GD1a IgG (1:400) and anti-GD1b IgG (1:200). A diagnosis of GBS was established. The patient underwent five sessions of plasma exchange and was treated with dexamethasone. His condition gradually improved, with recovery of muscle strength to grade 4+ in the arms and grade 3+ in the legs being observed. At the 3-month follow-up, the patient had near-complete recovery.

#### Case 2

A 50-year-old male was initially diagnosed with MM at an external hospital in December 2021 and presented with chest and rib pain, along with anemia. He received one cycle of the VD regimen (bortezomib and dexamethasone). In January 2022, he was admitted to Yidu Central Hospital of Weifang due to a pulmonary infection. The patient subsequently received a second cycle of VRD (bortezomib, lenalidomide, and dexamethasone) in February 2022, followed by a third cycle on March 18, 2022. On March 26, he developed diarrhea. On March 29, a neurological examination revealed decreased muscle strength, with both proximal and distal muscles graded as III in the arms and IV in the legs. On neurological examination, deep tendon reflexes were absent in both upper and lower limbs, including the biceps, triceps, patellar, and Achilles tendon reflexes. Notably, muscle atrophy and fasciculations were absent. Babinski sign was negative bilaterally. Sensory examination revealed intact proprioception, with joint position, movement and vibration sense preserved in the upper and lower limbs. Assessment of balance and Romberg test could not be performed due to severe muscle weakness. Light touch, pain, and temperature sensation were relatively preserved. On the fourth day after symptom onset, NCSs (Supplementary Table 1) indicated peripheral neuropathy involving both legs. Needle EMG examination (Supplementary Table 2) showed reduced voluntary recruitment, consistent with impaired neural activation rather than primary myogenic damage. CSF analysis revealed albuminocytological dissociation, with an elevated protein concentration of 928 mg/L (150–450 mg/L) and a normal cell count of 4 cells/μL (0–5 μL). Following neurology consultation, the patient was diagnosed with Guillain–Barré syndrome based on the clinical presentation (acute flaccid paralysis of all four limbs), disease course, and cerebrospinal fluid findings of albuminocytologic dissociation, fulfilling the Brighton Collaboration criteria for Level 2 diagnostic certainty. The patient was treated with IVIG at a dosage of 0.4 g/kg/day for 3 consecutive days. However, no improvement in limb muscle strength was observed. The patient subsequently chose to discontinue further treatment.

#### Case 3

A 67-year-old female presented to Yidu Central Hospital of Weifang in September 2024 and was diagnosed with MM (IgA-kappa subtype, Durie-Salmon stage II A, ISS stage I, mSMART 4.0 high-risk). Cytogenetic abnormalities included P53/CEP17 deletion, RB1 deletion, 1q21 amplification, and MYC rearrangement. The patient sequentially received four cycles of chemotherapy, including a VD cycle, two VRD cycles, and a VCD (bortezomib, cyclophosphamide, and dexamethasone), cycle. A treatment response was defined as the minimal response. On December 25, 2024, she was diagnosed with a pulmonary infection. The fifth cycle of VRD was initiated on January 3, 2025. On January 13, the patient developed recurrent diarrhea. By January 17, she had experienced urinary retention and was unable to stand. Post-void residual volume was measured at 1,190 mL, indicating severe bladder atony. The neurological symptoms included bilateral foot and arm numbness and tingling, with both proximal and distal muscle strength being graded as III in all four limbs. Sensory examination revealed decreased pinprick sensation, while temperature sensation was preserved. Neurological examination revealed intact proprioception, with preservation of joint position, movement and vibration sense in both upper and lower limbs. Evaluation of balance, including Romberg test, was not feasible because of severe muscle weakness. Deep tendon reflexes were absent in both upper and lower limbs, including the biceps, triceps, patellar, and Achilles tendon reflexes. Neurological examination revealed no muscle atrophy or fasciculations. Babinski sign was negative bilaterally. On January 25, MRI scans of the brain, as well as the cervical, thoracic, and lumbar spine, revealed vertebral compression and flattening at T7 and T9. On the seventh day after symptom onset, NCSs (Supplementary Table 1) indicated peripheral neuropathy with predominant axonal damage and no electrophysiological evidence of demyelination. Needle EMG examination (Supplementary Table 2) showed reduced voluntary recruitment, consistent with impaired neural activation rather than primary myogenic damage. The serum levels of folate and vitamin B12 were observed to be within normal limits. CSF analysis revealed albuminocytological dissociation, with an elevated protein concentration of 1,128 mg/L (150–450 mg/L) and a normal cell count of 3 cells/μL (0–5 μL). Following a neurology consultation, GBS was diagnosed. The patient was immediately started on IVIG for 5 days. The diarrhea resolved by approximately February 13, with normalization of bowel movements being observed. On February 19, the patient was switched to the DKD regimen (daratumumab, carfilzomib, and dexamethasone). By March 5, spontaneous urination had resumed. On approximately March 13 (about 1.5 months after initiation of IVIG), the tingling sensations in both feet and arms had markedly improved. Neurological examination revealed distal upper limb strength at grade III, proximal upper limb strength at grade V, and distal lower limb strength at grade IV.

### Clinical characteristics

A total of 33 patients (including 21 males and 12 females) were included in the final analysis (Supplementary Table 3). The median patient age was 67 years (range: 45–87 years). With respect to the immunoglobulin subtype, 1 patient exhibited the IgG type (light chain involvement not provided), 11 patients exhibited the IgG-κ type, 3 patients exhibited the IgG-λ type, 7 patients exhibited the IgA-κ type, 4 patients exhibited the IgA-λ type, and 3 patients exhibited the light chain λ type. For 4 patients, the involved immunoglobulin subtype was not provided. All of the patients received bortezomib-based therapy. The median cumulative dose of bortezomib at the time of GBS onset was 15.6 mg/m^2^ (range: 1.3–32.5 mg/m^2^). Among these patients, 25 developed GBS with a cumulative bortezomib dosage of ≤15.6 mg/m^2^. The details of these patients are provided in Table 1. Moreover, the electrophysiological and CSF findings in our cohort are summarized in Supplementary Table 3. Notably, 85% of the patients exhibited sensory-motor NCS abnormalities, whereas 76% demonstrated albuminocytologic dissociation.

**Table 1:** Characteristics of patients with GBS during V therapy.

Variables	No (*N* = 33)
Age	67 years (45–87)
Sex, *n*	
Female	12
Male	21
Ig type, *n*	
IgG-κ	11
IgG-*λ*	3
IgA-κ	7
IgA-λ	4
λ	3
NA	4
IgG	1
Cumulative V dosage	15.6 mg/m^2^ (1.3–32.5)

GBS, Guillain Barre syndrome; V, bortezomib.

### Diagnosis of GBS

GBS typically presents with an acute onset and a monophasic course, which stabilizes within 2–4 weeks. The core symptom involves limb weakness (with or without the involvement of sensory or autonomic nerves). CSF analysis typically reveals albuminocytologic dissociation. NCSs can reveal demyelinating lesions or axonal damage (depending on the clinical subtype of GBS) with the involvement of motor and/or sensory nerves. In Case 1, strongly supportive serological evidence for GBS (positive anti-GD1a/GD1b IgG antibodies) were exhibited, whereas Cases 2–3, clinical and electrophysiological criteria were primarily used. This scenario the spectrum of diagnostic certainty in our cohort. In two-thirds of cases, GBS is preceded by an event such as upper respiratory tract infection, diarrhea, or cytomegalovirus (CMV) reactivation within 6 weeks. Certain surgeries may also be associated with the development of this disorder. In this study, 13 patients reported whether they had experienced preceding events before the diagnosis of GBS. Among these patients, 5 patients did not experience any preceding events. Among the 8 patients with preceding events, the following events were noted: 1 patient with IgA-κ type MM developed GBS after experiencing a pulmonary infection accompanied by diarrhea; 1 patient with the *λ* light chain type experienced CMV reactivation before the onset of GBS; 1 patient without a specified Ig subtype developed GBS following a pulmonary infection with diarrhea; 1 patient with the IgA-*λ* type underwent percutaneous vertebroplasty (PVP) surgery prior to GBS onset; 1 patient with the IgA-λ type underwent PVP surgery and received autologous stem cell transplantation (ASCT), followed by the occurrence of an upper respiratory tract infection before the development of GBS; 1 patient with the IgG-κ type developed GBS after undergoing ASCT; and 1 patient with the IgG-κ type developed GBS following diarrhea.

NCSs revealed that 29 patients had both sensory and motor nerve damage, 2 patients had isolated sensory nerve damage, and 1 patient had isolated motor nerve damage. Among the 16 patients with demyelinating lesions, 8 patients exhibited axonal damage, 6 patients demonstrated combined demyelination and axonal damage, 1 patient demonstrated a loss of neurogenic control, and 1 patient exhibited neuronal damage. For 1 patient, no information on axonal or myelin involvement was provided. The details of the NCSs are provided in Table 2.

**Table 2:** Diagnostic of GBS.

Variables	No (*N* = 33)
Precipitating factors, *n*	
Infection	5
ASCT	1
PVP + Infection + ASCT	1
PVP	1
NA	20
No	5
CSF examination, *n*	
albumino-cytologic dissociation	23
No	3
NA	7
NCS functional impairment findings, n	
Sensory and motor nerve damage	30
Sensory nerve damage	2
Motor nerve damage	1
NCS types of injury, *n*	
Demyelinating damage	16
Axonal damage	8
Demyelination and axonal damage	6
Loss of neurogenic control	1
Neuronal damage	1
NA	1

ASCT, Autologous Stem Cell Transplantation; PVP, Percutaneous vertebroplasty; NA, Not Available; NCS, Nerve conduct study.

### Treatment and prognosis

In this study (Supplementary Table 3), following the onset of GBS, all but 1 patient (who experienced spontaneous remission) received active interventions, including the discontinuation of bortezomib, intravenous immunoglobulin (IVIG) infusion, plasma exchange, and neurological rehabilitation. It is important to note that IVIG and plasmapheresis are established first-line therapies for GBS. In our cohort, which includes 30 cases from the literature review and 3 cases from our center, IVIG (with or without corticosteroids and/or plasma exchange) was administered in all confirmed GBS cases, supporting this treatment approach (Table 3). With the exception of 1 patient who experienced progressive neurological deterioration, most of the patients achieved varying degrees of neurological recovery within weeks to months. Among the 24 patients who had data on recovery time, the shortest duration was 3 weeks, and the longest duration was 21 months. All of these patients exhibited at least partial improvements in motor function, with a median recovery time of 4.5 months (range: 3 weeks to 21 months). However, by the end of the follow-up period, only 5 patients had achieved complete neurological recovery. The details regarding treatment and prognosis are provided in Table 3.

**Table 3:** Treatment and prognosis of GBS.

Variables	No (*N* = 33)
GBS treatment, *n*	
PEX	2
IVIG	18
IVIG + PEX	2
PEX + Steroid	1
Steroid	2
IVIG + Steroid	2
NA	3
No treatment	3
GBS prognosis, *n*	
Complete neurological recovery	5
Partial improvement in motor function	21
Death due to GBS	1
Death due to other causes	2
NA	3
Worsening	1
Median recovery time	4.5 months (0.75 to 21)

PEX, Plasma exchange; IVIG, Intravenous immunoglobulin.

## Discussion

Proteasome inhibitors (such as bortezomib) have become among the cornerstone therapies for newly diagnosed or relapsed/refractory MM. Common adverse effects of bortezomib include thrombocytopenia, diarrhea, and peripheral neuropathy. Bortezomib-induced peripheral neuropathy (BIPN) primarily affects unmyelinated small nerve fibers and presents as a sensory, axonal injury (5). Clinically, BIPN manifests as a stocking-glove distribution of numbness, paresthesia, and neuropathic pain. However, a minority of patients may develop autonomic or motor neuropathies. The involvement of the motor system typically presents as muscle weakness, muscle atrophy, and diminished or absent tendon reflexes (6), thereby significantly impairing patients’ quality of life, increasing the healthcare burden, and increasing the risk of medical disputes. Based on literature reports and adverse event data, the European Medicines Agency (EMA) issued a warning in 2021 suggesting a potential association between bortezomib use and the occurrence of GBS.

BIPN, which is primarily characterized by sensory nerve damage, is a dose-dependent type of condition. The incidence of BIPN increases significantly when the cumulative dose of bortezomib reaches 16–26 mg/m^2^, with a plateau phase typically being observed after five treatment cycles (6). In our study of 33 patients, we observed that motor nerve involvement could occur as early as 6 days after the initial bortezomib administration (3). At the time of onset, the median cumulative dose of bortezomib was observed to be 15.6 mg/m^2^ (Supplementary Table 3), and most of these patients were ultimately diagnosed with GBS. The underlying mechanism of bortezomib-induced GBS is likely immune-mediated (7), as evidenced by the lower cumulative doses of bortezomib that are required to trigger GBS. Although there have been isolated cases of spontaneous resolution of GBS after the discontinuation of bortezomib (8), the majority of patients do not experience recovery of motor function with drug cessation alone. Treatments such as IVIG therapy and/or plasma exchange often lead to significant improvements in motor nerve function in most patients.

Bortezomib is commonly used in combination regimens with other antimyeloma agents that act by different mechanisms. These agents include immunomodulatory drugs (IMiDs), such as thalidomide and lenalidomide (Supplementary Table 3). Concomitant medications and the affected immunoglobulin (Ig) subtype may collectively contribute to the development of GBS in patients with MM treated with bortezomib. Among the 33 patients included in this study, the combination of bortezomib and thalidomide was more likely to induce GBS in patients with IgG-type myeloma, whereas the combination of bortezomib and lenalidomide appeared to be more commonly associated with GBS in those patients with IgA-type myeloma.

Infection is a well-known preceding event of GBS. In our study, 13 patients provided information regarding potential preceding events prior to the onset of GBS (Supplementary Table 3). Among these patients, five reported no identifiable antecedent events. Among the 8 patients who reported such events, the main triggers included respiratory or pulmonary infections with or without diarrhea, cytomegalovirus (CMV) reactivation, percutaneous vertebroplasty (PVP), and ASCT. These preceding events are temporally associated with GBS; however, their causal relationship remains unclear. Notably, among the 4 patients with preceding events involving infections, three had IgA-type myeloma. Immunoglobulin A (IgA) is primarily found on mucosal surfaces and in secretions such as those of the respiratory and gastrointestinal tracts, where it constitutes the first line of defense against pathogens. In patients with IgA-type myeloma, normal IgA synthesis is suppressed, thereby leading to an increased risk of respiratory and gastrointestinal infections. Furthermore, lenalidomide therapy is more frequently associated with neutropenia and infection (9). Spinal surgery may represent another potential risk factor for the development of GBS (10, 11). In our cohort, 2 patients underwent PVP prior to the onset of GBS. Additionally, ASCT has been implicated in the pathogenesis of GBS (12, 13), and 2 patients in our study had a history of ASCT.

In addition to motor nerve involvement, GBS can also affect sensory and autonomic nerves. Due to its nonspecific clinical manifestations, differential diagnosis is essential. POEMS syndrome (14) is a constellation of various symptoms, including polyneuropathy, organomegaly, endocrinopathy, monoclonal plasma cell disorder (involving the M protein), and skin changes. Both POEMS syndrome and GBS are characterized by peripheral neuropathy; however, POEMS-related neuropathy typically presents as a chronic or subacute onset, with symmetric distal sensorimotor polyneuropathy and rare autonomic involvement being observed. Moreover, it is often accompanied by markedly elevated vascular endothelial growth factor (VEGF) levels, multisystem involvement, and characteristic sclerotic bone lesions. In contrast, GBS demonstrates an acute onset and manifests as acute symmetrical flaccid paralysis, which usually involves autonomic nerves and typically lacks multisystem involvement. Peripheral nerve damage may also occur secondary to amyloidosis in patients with MM (15, 16); in such cases, skin biopsy is often required for a definitive diagnosis. Notably, bortezomib itself has been reported to cause demyelinating neuropathy, as documented by Thawani et al. (3), which should be considered when differential diagnoses ae being evaluated. During the course of bortezomib treatment in multiple myeloma, if GBS is suspected, prompt diagnosis and differential evaluation are crucial. The diagnosis of GBS should adhere to established guidelines, which highlight typical features including acute ascending paralysis, albuminocytologic dissociation, and electrophysiological evidence of demyelination or axonal injury as demonstrated by NCS. For patients with confirmed GBS, the early initiation of immune treatments such as intravenous immunoglobulin (IVIG) (17) and plasma exchange can significantly improve patient outcomes.

## Conclusion

Given the profound impacts of GBS on quality of life and prognosis in MM patients, regular neurological assessments are warranted during bortezomib therapy (even in asymptomatic individuals). These evaluations should include regular clinical neurological examinations (such as sensory/motor function assessments), with prompt referrals for nerve conduction studies (NCSs) and cerebrospinal fluid analysis being performed if neurological symptoms emerge (2, 18). Furthermore, when considering the rarity of GBS and its devastating effects on patients’ quality of life, economic burden, and overall survival, multicenter collaborative studies are needed. Such efforts would facilitate the accumulation of more cases to identify risk factors, elucidate potential pathophysiological mechanisms, establish effective early warning strategies, and explore preventive measures for bortezomib-associated GBS in multiple myeloma patients.

Finally, this study has several limitations, including its small sample size and retrospective design, which may limit the generalizability of the findings. Additionally, there is a potential publication bias favoring the inclusion of more severe or atypical cases of this disorder. Another limitation is that contrast-enhanced MRI was not performed in this patient, and thus nerve root enhancement could not be assessed. Future studies may include contrast-enhanced MRI to further support the diagnosis of GBS.

## Data Availability

The original contributions presented in the study are included in the article/Supplementary material, further inquiries can be directed to the corresponding author.
